# Feasibility and Safety of Three-Port Laparoscopic Cholecystectomy Compared to Four-Port Laparoscopic Cholecystectomy

**DOI:** 10.7759/cureus.19979

**Published:** 2021-11-29

**Authors:** Mohd Yunus Shah, Umeshraj Somasundaram, TRVRaju Wilkinson, Nitin Wasnik

**Affiliations:** 1 Department of Surgery, NKP Salve Institute of Medical Sciences and Research Centre, Nagpur, IND; 2 Department of General Surgery, Melmaruvathur Adhiparasakthi Institute of Medical Sciences and Research, Chengalpet, IND

**Keywords:** three-port technique, four-port technique, cholelithiasis., minimally invasive surgery, laparoscopic cholecystectomy

## Abstract

Background

The standard four-port laparoscopic cholecystectomy (LC) is the gold standard procedure. The various clinical trials and reports in the literature have suggested that the three-port technique LC can be done safely with acceptable results.

Still, that the three-port LC offers any added benefits to the patient is a controversial issue especially in view of safety and feasibility. In this study, we report the experience of three-port LC compared to four-port LC technique, its safety, feasibility and outcomes.

Materials and methods

A prospective randomized study was conducted between two groups which included 165 cases - 93 patients were included in three-port LC (Group A) and 72 patients in four-port LC (Group B). Operative time, intraoperative complications, postoperative pain, length of hospital stay, analgesics requirement, conversion to open and return to normal activities were parameters of evaluation.

Results

Demographic data was comparable in both the groups. Three-port LC Group A had lesser post-operative pain and analgesics requirements. The mean postoperative pain visual analogue scale (VAS) score on day 1 was (4.16 and 6.24), on day 7 was (1.26 and 1.81) in three-port group and in four-port LC group, respectively. The mean days of analgesics requirement were 2.56 days and 4.21 days among three-port group and four-port group, respectively

Length of hospital stay was less and returning to work was early in three-port group. There was no statistical difference in operative time. The mean operative time among three-port LC group A and four-port LC group B was 36+/-8.6 minutes (30-68) and 39+/-7 minutes (30-90), respectively. The overall outcomes were comparable to four-port LC.

Conclusion

Three-port LC is a feasible and safe procedure for LC with satisfactory outcomes like lesser postoperative pain, postoperative stay and less scars, when performed by experienced hands, especially in acute cholecystitis. The use of fourth port should be done when required in a difficult situation.

## Introduction

Standard four-port laparoscopic cholecystectomy (LC) is considered to be a gold standard technique for cholecystectomy [1, 2].

Various modifications have been done in the four-port laparoscopic cholecystectomy like decreasing the number and size of the ports to reduce the postoperative pain and better cosmetic results [3-6]. The use of the fourth port has been questioned by many surgeons and several studies in the literature have reported that three-port LC can be performed safely as it is a feasible technique with comparable outcomes [5,7-9].

These studies have mentioned that three-port LC outcomes need to be re-evaluated by other large sample studies.

These modifications have shown reduced postoperative pain and less use of analgesics. Some surgeons have reported the use of two ports and mini-instruments for doing LC [4,10,11].

The present study was conducted to compare the feasibility and safety of three-port laparoscopic cholecystectomy as compared to four-port LC. The outcome was measured based on operative time, intraoperative complications, conversion to four-port or open procedure, postoperative pain by visual analogue scale (VAS), length of hospital stay, resuming normal activities and overall satisfaction of the patient.

## Materials and methods

A randomized prospective study was carried out at a tertiary care teaching hospital in central India, between August 2017 and August 2020. A total of 165 patients who required laparoscopic cholecystectomy between the age group 18-75 years were included in the study after the Narendra Kumar Prasadrao Salve ethical committee approval. Informed consent was taken from all the patients included in the study. Patients were divided into two groups - three-port LC (Group A) had 93 patients and four-port LC (Group B) had 72 patients. Operative time, complications, postoperative pain, length of hospital stay, analgesics requirement, conversion to open and return to normal activities were evaluated in both the groups.

The data regarding demographic characteristics, clinical history, examination findings, and investigations, intraoperative and post-operative findings was collected using pre-validated, semi-structured, standard case record proforma.

**Figure 1:**
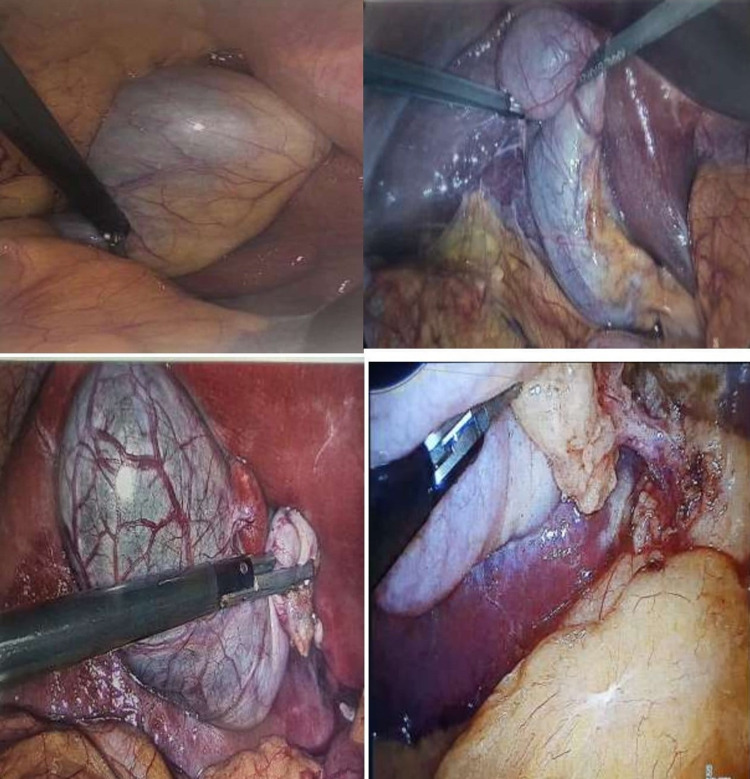
Laparoscopic views of three-port laparoscopic cholecystectomy.

**Figure 2:**
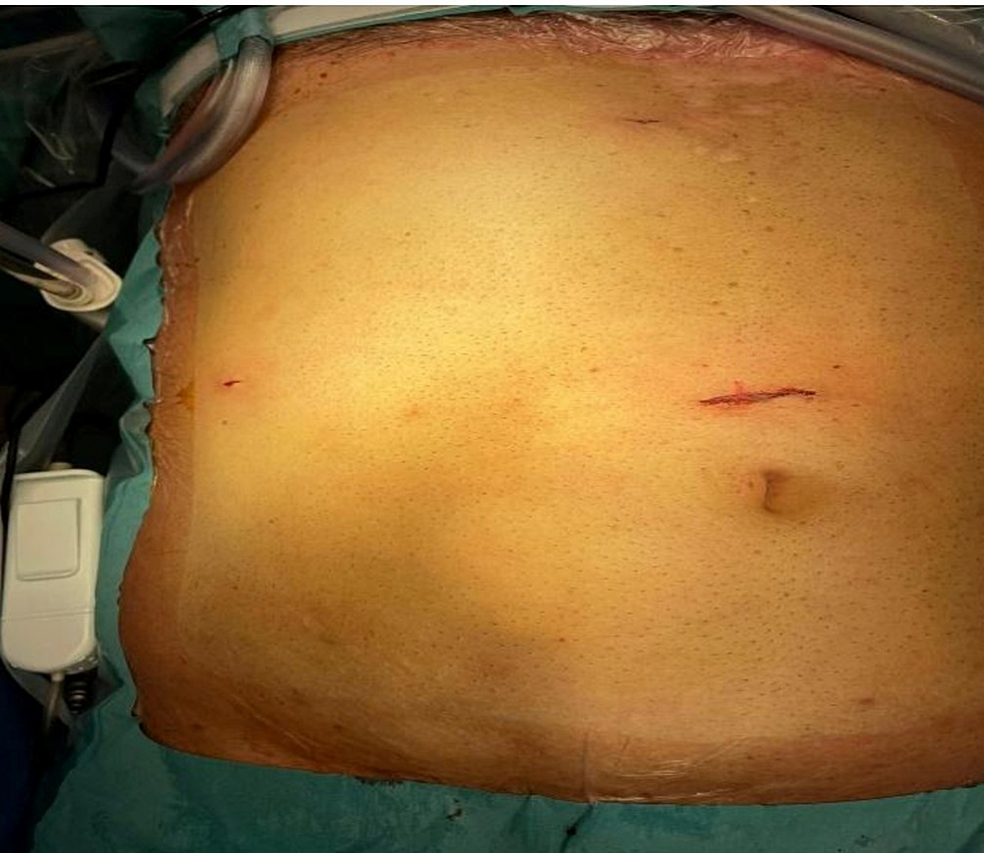
Ports position in three-port laparoscopic cholecystectomy. Three-port laparoscopic cholecystectomy - a 10-mm supra-umbilical camera port, two 5-mm working ports in epigastric and right hypochondrium region.

The three-port technique was performed by creating pneumoperitoneum using Veress needle technique through a supra-umbilical stab incision through which a 10-mm camera port (Xcel port from Ethicon Endo-Surgery, Cincinnati, OH) was introduced. An operating telescope (Karl Storz Image 1 three-chip system, Karl Storz SE & Co. KG, Tuttlingen, Germany) was introduced from the supra-umbilical camera port. Another 5-mm trocar (Endopath Xcel port dilating tip, Ethicon Endo-Surgery) was inserted 3 cm below the xiphisternum, and after evaluation of the gall bladder position and anatomy, a 5-mm working port (Endopath Tristar trocar, Ethicon, Inc., Raritan, NJ) was introduced at the right hypochondrium anterior axillary line 3 cm below the subcostal margin. The operating surgeon and assistant stood on the left side of the patient while the staff nurse stood on the right side of the patient. The monitor, insufflation and light source system were kept on the right side of the patient. The gall bladder infundibulum was held and retracted towards the right upwards direction from the right subcostal 10-mm port. A 5-mm epigastric port was used for Calot’s triangle dissection using blunt and sharp hook intermittently. After doing the posterior dissection, a critical view of safety was seen. Cystic artery and cystic duct were dissected in Calot’s triangle. The cystic duct was then clipped with a 5-mm clip applicator and divided followed by the cystic artery. Intraoperative cholangiogram (IOC) if indicated was performed through the 5-mm epigastric port by using a fluoroscope system. After the dissection of the gall bladder from the gall bladder fossa in proper plane, the gall bladder was separated. The specimen was retrieved from the 10-mm supra-umbilical port using the 5-mm telescope from the epigastric port under vision. After achieving the proper hemostasis, trocars were removed under vision. Port sites were sutured with Vicryl 3-0 cutting needle suture (Figures 1, 2).

The four-port LC was performed using the fourth port at the right subcostal region introduced as per the convenience and position of the gall bladder for retraction of fundus of the gall bladder. The rest of the procedure was the same as three ports LC.

After surgery patients were shifted to the postoperative care unit. After complete stabilization, the patients were transferred to the ward where analgesics were administered (paracetamol and/or diclofenac) unless allergies or specific contraindications were noted. Patients received their analgesics according to their pain ratings measured by nursing staff using the visual analogue scale. The total amount of analgesia required by each patient was calculated over 48 hours post-surgery or till the patient was discharged. The patient was discharged after the consensus in the surgical team, nursing staff and the patient regarding the recovery. Ports dressing was checked for any bleeding or hematoma. Post-discharge, the patients were prescribed antibiotics and analgesics with advice for follow-up in OPD after one week.

Statistical analysis

Continuous data is presented in form of mean ± SD and compared using independent t-test whereas categorical data is presented in frequency (%) and compared using Chi-square test. Statistical software named “MedCal - 12.2.1” was used for analysis. Significance is set at 5% in this study.

## Results

In the present study demographic characteristics among Group A three-port LC (n=93) and Group B four-port LC (n=72) were compared. The mean age of the study subjects was 42.52 +/- 3.7 years in Group A and 46.37 +/- 15.01 years in Group B (p-value: 0.018). On gender-wise distribution of the study subjects, it was observed that the majority were females in both study groups, however, in Group A ratio of females was greater (p-value: 0.017).

In the current study preoperative diagnosis was assessed. It was observed that the majority had symptomatic cholelithiasis (81.72% and 86.11% in either group), followed by acute cholecystitis (15.05% and 12.5%, respectively) and gall bladder polyps (2.35% and 1.38%, respectively). In this study, previous endoscopic retrograde cholangiopancreatography (ERCP) due to choledocholithiasis was done among 5.38% and 9.72% patients in both Group A and Group B, respectively. The difference was not statistically significant (p-value: 0.445).

On the assessment of previous upper abdominal surgery among the study subjects, no statistically significant difference was observed among both the groups (p-value: 0.160). Table 1 shows the comparison of clinical data in two groups of patients.

**Table 1:** Comparison of clinical data in two groups. LC - Laparoscopic cholecystectomy; ERCP - Endoscopic retrograde cholangiopancreatography # indicates Chi-square value * indicates t-value

Variables		Group A three-port LC (n=93)	Group B four-port LC (n=72)	Test value	P-value
Age Mean +/- SD		42.52 +/- 3.7	46.37 +/- 15.01	-2.384*	0.018
Gender	Female	67 (72.04%)	38 (52.75%)	5.703#	0.017
	Male	26 (27.95%)	34 (47.25%)		
Preoperative Diagnosis	Chronic Cholecystitis	76 (81.72%)	62 (86.11%)	0.848#	0.654
	Acute Cholecystitis	14 (15.05%)	9 (12.5%)		
	Gall bladder polyps	3 (2.35%)	1 (1.38%)		
Previous ERCP due to Choledocholithiasis		5 (5.38%)	7 (9.72%)	0.583#	0.445
Previous upper abdominal surgery		3 (3.23%)	6 (8.33%)	1.975#	0.160

In the present study, intraoperative and postoperative variables were studied as shown in Table 2. The mean operative time among three-port LC Group A and four-port LC Group B was 36 ± 8.6 minutes (30-68) and 39 ± 7 minutes (30-90), respectively. The operative time was relatively lesser in three-port group as the time taken for fourth-port insertion and closure was reduced (p-value: 0.019). The operative time was longer in patients with previous upper abdominal surgeries, history of cholecystitis and intraoperative difficult Calot’s triangle dissection due to adhesions.

**Table 2:** Intraoperative and postoperative variables in both the groups. # indicates Chi-square value * indicates t-value SD - Standard deviation; LC - Laparoscopic cholecystectomy

Variables	Group A three-port LC (n=93)	Group B four-port LC (n=72)	Test value	p-value
Operative time (min) Mean +/- SD (range)	36 +/- 8.6 (30-68)	39 +/- 7.4 (30-90)*	-2.360	0.019
Signs of acute cholecystitis	17 (18.28%)	12 (16.67%) #	0.004	0.949
Calot’s triangle adhesions	7 (7.52%)	6 (8.33%) #	0.010	0.919
Gall bladder perforation during dissection	7 (7.52%)	3 (4.16%) #	0.323	0.570
Stone spillage	5 (5.37%)	4 (5.55%) #	0.087	0.767
Bleeding due to clip slippage	5 (5.37%)	3 (4.1%) #	0	0.994
Anatomical variations	5 (5.37%)	6 (8.33%) #	0.194	0.659
Drain	7 (7.53%)	5 (3.6%) #	0.025	0.873
Port site bleeding	2 (2.15%)	3 (4.17%) #	0.085	0.771
Conversion from three-port to four-port LC	--	4 (4.3%) #	NA	NA
Conversion to open surgery	1 (1.08%)	2 (2.78%) #	0.050	0.823

Signs of acute cholecystitis were not significantly different among both the groups. Gall bladder perforation during dissection and stone spillage were reported more in three-port LC group as compared to four-port LC group. There was no statistically significant difference found in Calot’s triangle adhesions for both the groups.

Anatomical variations were noted in five patients (5.37%) and six patients (8.33%) in three-port LC Group A and four-port LC Group B, respectively (p-value: 0.45). Conversion to open surgery was observed among one patient (1.08%) and two patients (2.78%) in Group A and B, respectively (p-value: 0.823). Drain was placed among seven patients (7.53%) and five patients (3.6%) in Group A and B, respectively (p-value: 0.87). Port site bleeding was reported among two patients (2.15%) and three patients (4.17%) in Group A and B, respectively (p-value: 0.771). Conversion to four ports from three-port LC was observed among four patients (4.3%).

The mean postoperative pain VAS score on day 1 was 4.16 and 6.24, day 7 1.26 and 1.81 in three-port group and in four-port LC group, respectively (p-value <0.001). Mean days of analgesics requirement were 2.56 days and 4.21 days among three-port group and four-port group, respectively (p-value <0.001). Mean hospital stay duration was 3.12 days and 4.86 days among three-port group and four-port group, respectively (p-value < 0.001 ). Mean days of return to normal activities were 4.5 days and 6.2 days among three-port group and four-port group, respectively. Mean satisfactory score after seven days was 7.6 and 6.4 among three-port group and four-port group, respectively (p-value < 0.001). Port sites infection was observed among one patient (1.07%) and two patients (2.14%) among three-port group and four-port group, respectively. Postoperative jaundice was observed in one patient (1.08%) in three-port group.

In the current study, we assessed the reasons for conversions of patients from three-port LC to four-port LC or to open procedure as shown in Table 3. It was observed that adhesions and bleeding were the commonest reasons for conversions from three-port LC to four-port LC in two (2.14%) patients, followed by bile spillage, difficult Calot’s Triangle dissection, and abnormal anatomy and clip displacement in one patient (1.07%).

**Table 3:** Reasons for conversions from three-port LC to four-port LC. LC - Laparoscopic Cholecystectomy.

Conditions	Number of subjects (n=165)
Adhesions	2 (2.14%)
Bleeding	2 (2.14%)
Difficult Calots Triangle dissection	1 (1.07%)
Abnormal anatomy	1 (1.07%)
Clip displacement	1 (1.07%)
Total	7 (4.2% )

## Discussion

Four-port laparoscopic cholecystectomy is the gold standard procedure for LC. The aim of the laparoscopy procedure includes decreased pain, improved cosmetic results and decreased duration of hospital stay compared to laparotomy. Over a period of time, LC has been modified and developed in many ways including reduction in size and number of ports for the benefit of the patients as reported in the literature [3-6,12,13].

Four-port LC is most commonly used, as it has better anatomic views and is easier to learn. The use of the fourth port in LC has been questioned by many experienced surgeons and there are several literature reports which mention that the use of fourth port can be avoided and LC can be done with three ports without compromising patient’s safety [7, 8]. Cerci et al. [9] have reported in their study that three-port laparoscopic cholecystectomy is safe, economic and effective but does not reduce the postoperative pain and analgesics requirement.

A meta-analysis study by Sun et al. [14], which compared three-port laparoscopic cholecystectomy (LC) and four-port LC, reported that the operative time pain medications requirement, complications and length of hospital stay were not significantly different between the two.

The technique of LC is modified over a period of time to achieve better results. The fourth port has been used in LC to retract the gall bladder fundus for better visualization of biliary anatomy, Calot’s triangle and to achieve the critical view of safety during LC [15].

In this study comparison of outcome measures, feasibility and safety of three-port LC as compared to four-port LC in acute as well as chronic cholecystitis cases has been done. There were no bile duct injuries or any major complications in either of the patients. Port site bleeding/hematomas were seen less frequently in patients who underwent three-port LC, however, the difference was not statistically significant. The conversion rate, when compared with four-port LC, did not change as shown in the literature as well [16, 17].

In the present study, we also compared demographic characteristics among the two groups. The mean age of the study subjects was 42.52 ± 3.7 years in Group A and 46.37 ± 15.01 years in Group B (p-value: 0.018). In the gender-wise distribution, the majority of subjects were females in both study groups, however, in Group A proportion of females was greater (p-value: 0.017). In the current study, we assessed preoperative diagnosis, where symptomatic cholelithiasis (81.72% and 86.11% in either group), acute cholecystitis (15.05% and 12.5%, respectively) and gall bladder polyps (2.35% and 1.38%, respectively) were reported. Previous ERCP due to choledocholithiasis was done among 5.38% and 9.72% of study subjects in respective groups, and this observation was not statistically significant (p-value: 0.445). When previous upper abdominal surgery was assessed among the study subjects, significantly different observation among either study group was noted (3.23% and 8.33% in either group) (p-value: 0.160).

In our study, the mean operative time among three-port LC Group A was 36 ± 8.6 (30-68 min) and in Group B four-port LC it was 39 ± 7.4 (30-90 min). The operative time was relatively lesser but not significantly different (p-value -0.019) in three-port group. Similar durations of operative time were reported in various studies in the literature in comparison between three-port LC and four-port LC [14].

The operative time in three-port LC is comparatively shorter in comparison with four-port LC due to more time consumed in insertion and closure of fourth port in four-port LC which sometimes may need hemostasis. Moreover, in four-port LC the operator spends more time in adjusting the fourth port instrument used to retract the gall bladder fundus which is usually handled by the 2nd assistant or the nursing staff which is not there in three-port LC, where it's done by the surgeon himself.

Signs of acute cholecystitis were not significantly more among three-port LC group as compared to four-port LC group. Gall bladder perforation during dissection, bleeding due to slippage of clip and stone spillage were reported more among three-port LC group as compared to four-port LC group but not statistically significant. Calot’s triangle adhesions were not found to be significantly different in either group.

Anatomical variations were observed in five (5.37%) and six (8.33%) study subjects in Group A and B, respectively (p-value: 0.45). Conversion to open surgery was observed among one (1.08%) and two (2.78%) study subjects in Group A and B, respectively (p-value: 0.41). The drain was placed among seven (7.53%) and five (3.6%) study subjects in Group A and B, respectively (p-value: 0.87). Port site bleeding was reported among two (2.15%) and three (4.17%) study subjects in Group A and B, respectively (p-value: 0.771). Conversion to four-port from three-port LC was observed among four (4.3%) patients.

The mean postoperative pain VAS score on day 1 was 4.16 and 6.24, day 7 1.26 and 1.81 in three-port group and in four-port LC group, respectively. Mean days of analgesics requirement was 2.56 days and 4.21 days among three-port group and four-port group, respectively. Mean hospital stay duration was 3.12 days and 4.86 days among three-port group and four-port group, respectively, Mean days of return to normal activities was 4.5 days and 6.2 days among three-port group and four-port group, respectively. The mean satisfactory score at seven days was 7.6 and 6.4 among three-port group and four-port group, respectively.

As reported in other studies, the present study also showed comparable less postoperative pain, less analgesics requirements, less number of days of hospitalization and early resumption of work in three-port LC as compared to four-port LC [3, 7-8, 18].

However, there was no statistical difference in the postoperative pain and complications on day 7 in both the groups.

Port sites infection was observed among one (1.07%) and two (2.14%) patients among three-port group and four-port group, respectively. There was post-operative jaundice in one patient (1.08%) in three-port group which was managed by ERCP and bile duct stenting.

In the present study, we have assessed the conditions which required conversions from three-port LC to four-port LC or to open cholecystectomy which is rarely reported in the previous literature. Calot’s triangle adhesions due to severe acute cholecystitis and bleeding were the main reasons for the conversions. Four patients (4.3%) required conversion from three-port LC to four-port LC. One patient (1.08%) from three-port LC and two patients (2.78%) from four-port LC were converted to open cholecystectomy due to difficult Calot’s triangle dissection and bleeding, keeping the low threshold for conversion. Other reasons for conversions were bile spillage, abnormal anatomy and displacement of clip in one (1.07%) patient.

All the procedures were performed by two experienced surgeons. The conversion rates were on the higher side but not significantly different from the other studies, maybe because the low threshold for conversion was kept. There was no incidence of bile duct injury or any major complications [3, 13, 18-19].

After the introduction of LC, there were concerns regarding more rate of biliary injuries [20]. Various modifications have been done in LC, three-port LC is one of them showing comparable results and feasible technique for LC as reported in the different studies [7-8, 13].

Today in the era of minimally invasive surgery the main advantages of LC are significant less postoperative pain, less hospital stay, early resumption of routine activities and better cosmoses. Various studies in the literature have shown that all these can be achieved better by reducing the size and the number of the ports without compromising the patient’s safety [8, 21-24].

This study has the limitation that the sample size was not large enough. We had a larger rate of conversion to four-port as the threshold for conversion to convert to four ports or to open procedure was kept at low in the interest of patients' safety.

## Conclusions

In this study, we conclude that the use of three ports in LC is feasible and can be performed safely with acceptable and comparable outcome measures as in routine four-port LC. We can achieve better results with added advantages of minimally invasive surgery offered to the patients by using the three-port technique of LC without compromising the patient’s safety. It is advised to do a similar kind of prospective study with a larger sample size. However, it should be recommended that three-port LC be performed by experienced hands and keeping a low threshold for the fourth port insertion or open conversion whenever indicated in a difficult situation.
